# Evidence of mood states in reptiles

**DOI:** 10.1007/s10071-025-01973-y

**Published:** 2025-06-28

**Authors:** Tatjana Hoehfurtner, Anna Wilkinson, Sophie A. Moszuti, Oliver H.P. Burman

**Affiliations:** School of Natural Sciences, University of Lincoln, Lincoln, LN6 7DL UK

**Keywords:** Affective state, Mood, Cognitive bias, Reptile welfare, Sentience

## Abstract

There is increasing evidence that non-human animals experience ‘free-floating’ mood states, but such evidence is lacking in reptiles, hindering the debate as to their affective capacity and with subsequent implications for welfare. Here, we investigated the presence of a mood state in a species of reptile, the red-footed tortoise (*Chelonoidis carbonaria*), using a spatial cognitive judgement bias task — an approach reliably used to determine background mood — alongside their behavioural response in anxiety tests. Our results showed that, as found in mammals and birds, individuals kept in appropriate conditions showed an optimistic mood, approaching ambiguous locations more rapidly when these were positioned closer to a rewarded location. This finding was reflected in associations between cognitive bias performance and behaviour in the concurrent anxiety tests, with more optimistic individuals showing less anxious behaviour in response to novelty. These findings significantly extend contemporary knowledge of the affective and cognitive capacity of reptiles and have important implications, not only for informing the management of reptiles but also for furthering our understanding of phylogenetic pathways of affective state.

## Introduction

Animal welfare concerns are informed by evidence that a given species has the capacity to experience moods and emotions, influencing how we interact with them and guiding our expectations for their quality of life (Mason and Mendl 1993). Emotions are short-term states elicited by rewards and punishers (Rolls 2005), whereas moods are longer-term ‘free-floating’ states unattached to a specific object or event, reflecting background subjective experience (Mendl and Paul 2004). Evidence of such affective states (used here to incorporate both emotions and moods) plays an important role in the attribution of sentience – the capacity to experience valenced affective states (e.g. Browning and Birch 2020) — directly impacting welfare-related legislation (e.g. Animal Welfare (Sentience) Act 2022).

Affective states likely evolved as a means for animals to more effectively avoid harm and acquire desirable resources (Trimmer et al. 2013). Given such advantages, both emotions and moods should therefore be expected to occur throughout the animal kingdom (Dawkins 2000), although the degree to which different animal species are able to experience such states is likely to vary (Paul et al. 2005). Understanding the comparative affective capabilities of a species not only informs us about their welfare and conservation but, because affective states can influence cognition (e.g. cognitive biases in decision-making (Harding et al. 2004), it can also reveal valuable insights into variation in cognitive performance. For example, changes in affective state may underpin fluctuations in animal learning and memory (see Mendl 1999; Mendl et al. 2001), such that they perform poorly when distressed (e.g. Regolin et al. 1995) or better when provided with social support (e.g. de Franca Malheiros et al. 2021).

However, whilst research has demonstrated the presence of affective states in a range of different animal species across a variety of contexts, the evidence is far less clear for reptiles — despite the importance of filling this knowledge gap for improving captive-reptile welfare (Lambert et al. 2019; Warwick et al. 2023). Whilst behavioural complexity and impressive cognitive capabilities have been demonstrated in reptiles (e.g. Matsubara et al. 2017), this alone is not evidence of a capacity for sentience (see Learmonth 2020) — although intelligence can be associated with poor captive welfare (Mellor et al. 2021). Discrete emotions have been partly demonstrated in different reptile species, including negative physiological (‘emotional fever’, Cabanac and Gosselin 1993) and behavioural (Stockley et al. 2020) responses to handling, as well as changes in behaviour indicative of anxiety when exposed to novelty (Moszuti et al. 2017). Reptiles also actively seek out positive experiences (Burghardt 2013), as demonstrated during preference for high-value food rewards, for which they will also work harder (Soldati et al. 2017), and enriched environments (Hoehfurtner et al. 2021a, b; Rickman et al. 2025). However, reptiles do not appear to show other types of emotional response identified in mammalian and avian species, such as incentive contrast (Flaherty 1996; Freidin 2009), which has been taken to suggest that they might experience a comparatively narrow affective spectrum (Papini 2003) and that some affective states may have evolved later in the evolutionary timeline (Papini et al. 1995; Papini 2002).

When it comes to assessing affective state in animals, cognitive judgement bias tasks have become an established method, providing evidence for the presence of background mood (e.g. Harding et al. 2004) and stimulus-elicited emotional (e.g. Burman et al. 2009) states across the animal kingdom (Mendl et al. 2009; Bethell 2015; Lagisz et al. 2020). During these tasks, generalisation is impacted by the animals’ affective state, resulting in a shift that can go in either a positive or a negative direction indicative of affective valence. Cognitive bias tasks have never been used in a reptilian species and the technique offers an ideal opportunity to investigate, for the first time, whether the presence of a mood state can be observed in reptiles, informing the long-standing debate as to their affective capacity (e.g. Lambert et al. 2019; Learmonth 2020).

To assess this we used a correlational approach (e.g. Mendl et al. 2010) in which we included two types of behavioural anxiety test, a novel object test (e.g. Siviter et al. 2016) and a novel environment test (e.g. Moszuti et al. 2017), alongside a cognitive judgement bias task, and predicted that there would be associations between cognitive bias performance (identifying the presence of a long-term background mood state) and response to novelty (indicating short-term emotions), with those individuals that judge ambiguous stimuli more optimistically showing less anxious behaviour in response to novelty.

## Methods

### Subjects

We used red-footed tortoises (*Chelonoidis carbonaria*) (*N* = 15) with plastron lengths ranging from 10.3 to 24.3 cm. Subjects were group-housed in a humidified room maintained at approximately 28^o^C with a 12 h light/dark schedule (0700–1900). All tortoises were housed on a bark substrate, had access to basking spots, UV light, humid hides and shelter. Food was provided once a day for six days a week, and water *ad libitum*.

### Apparatus

**Fig. 1:**
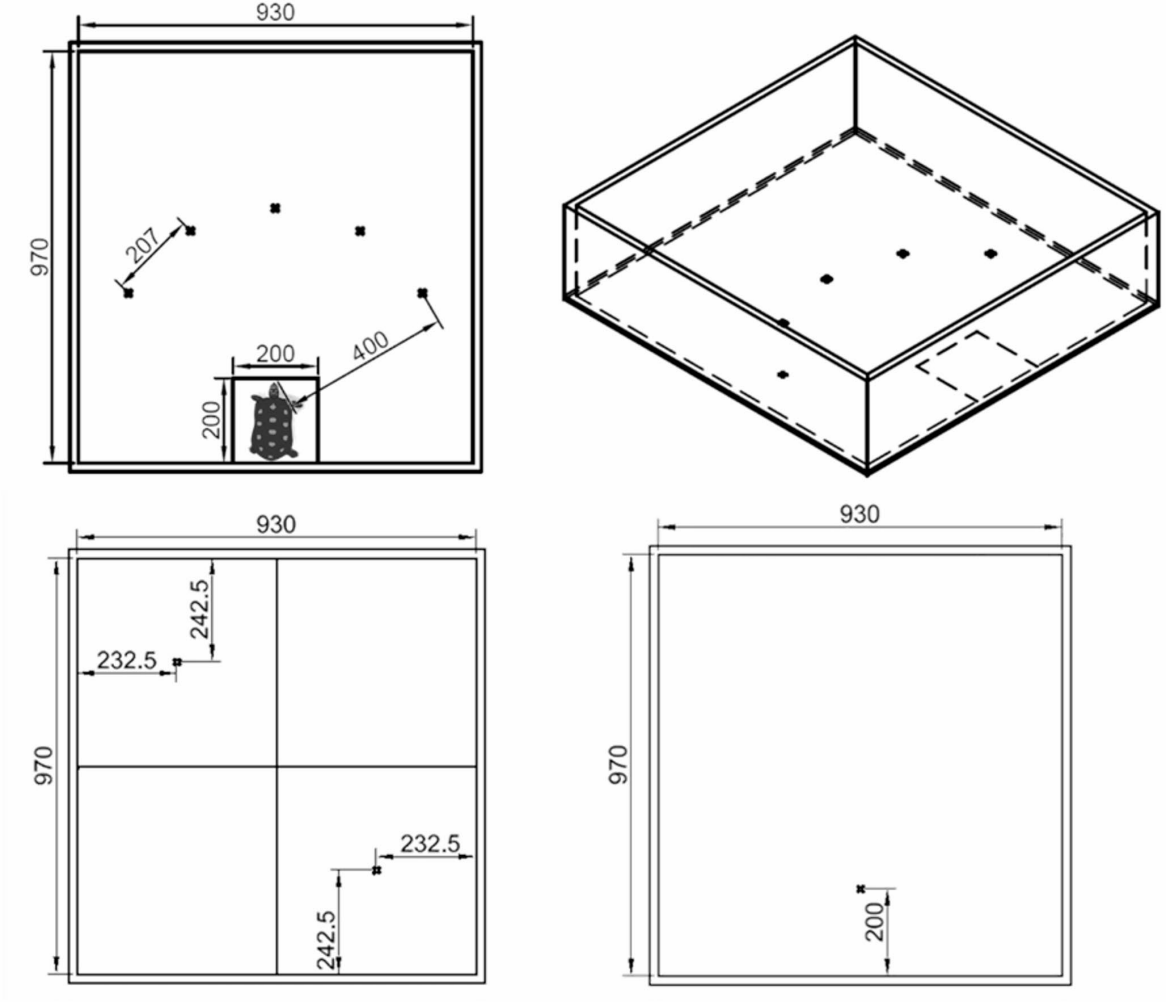
Experimental set up for cognitive bias tests (top), novel object test (bottom left), and novel environment test (bottom right). Measurements (mm) shown are for Arena (**A**) Arena B set up was the same except for distances. Arena B for the cognitive bias and novel object test measured ~ 150*170*62 cm. The start box in arena B for the cognitive bias tests was 35*35 cm and the bowl markers were 65 cm from the start box and 34 cm apart. For the novel object test the start position and novel object were 37.5*42.5 cm from the edge for Arena (**B**) Arena B for the novel environment test was ~ 135*170*62 cm and the start position was 35 cm from the edge. For the cognitive bias test crosses mark bowl locations and the tortoise in the box marks the starting location. [*For the novelty tests crosses mark start positions and object position.*]

The tortoises were assigned to one of two test arenas based on their size. The smaller tortoises (10.3–17.8 cm) were tested in an arena measuring 97*93*26 cm (Arena A) and the larger tortoises (18.4–24.3 cm) were tested in an arena arena measuring 150*170*62 cm (Arena B). A start position measuring 20*20 cm for Arena A and 35*35 cm for Arena B was marked on the floor within which the tortoises were placed at the start of each trial. Five food bowl locations were marked on the floor of each arena with a small cross. For Arena A, these were all 40 cm from the start box and 20.7 cm apart. For Arena B, these were all 65 cm from the start box and 34 cm apart (see Fig. 1). A small blue food bowl ~ 6.5 cm in diameter was used as the stimulus for the cognitive bias task and could be placed at any of the marked locations. All trials were video recorded, and the arenas were cleaned prior to each test.

### General design

As with other studies of cognitive bias (Mendl et al. 2010), we used a correlational approach to investigate links between individual performance in a cognitive bias task and response in a behavioural test. Specifically, we investigated whether there were correlations between cognitive bias performance and response to novelty, a measure that has been developed to assess anxiety in reptiles, including the red-footed tortoise (Moszuti et al. 2017). To do so we first exposed each of the fifteen subjects to cognitive bias tests, before going on to measure each individual’s response to two types of novel stimulus: (1) a novel object, and (2) a novel environment.

### Cognitive bias task

All tortoises received habituation trials in which they were placed in the arena, allowed to move around the arena and feed. Once the tortoises had eaten food for two habituation trials in a row, they were considered habituated and went on to the cognitive bias task. We adapted the established spatial cognitive bias go/no-go task (Burman et al. 2008; Mendl et al. 2010). Tortoises were trained to approach a bowl positioned at a positive (P) location and avoid it when positioned at a negative (N) location. The actual locations (positive/negative) were counterbalanced between subjects. Latency (s) to reach the bowl (defined as when the tortoise was within 5 cm of the bowl) was recorded for each trial. On arrival at the P location a food reward (rocket) was placed into the bowl, avoiding any possibility of olfactory cues being used to inform response (Soldati et al. 2017). No food reward was given upon arrival at the N bowl location. Subjects failing to reach the bowl (either N or P) within the 120s trial duration were ascribed a latency of 120s. Training trials were carried out with the bowl location occurring in a specified order (P, P, N, N, P) for the first 5 trials and then pseudo-randomly, with no more than two P or N trials consecutively and with the first trial of the session always starting with P. There were 9 trials per session, with an inter-trial interval of 2 min, and one session per day. The criterion for progressing from training to test trials was achieved when the tortoise was faster to reach the bowl in each of its last three P trials than in each of its last three N trials in six consecutive training trials (Burman et al. 2011) after completing a minimum of 36 trials. During testing (one session of nine test trials per day, for three days), all tortoises were exposed to three unrewarded ambiguous intermediate locations (near negative NN, middle M and near positive NP), experiencing each three times in different orders interspersed between two presentations of the reference P and N locations (e.g. P, N, M, P, N, NN, P, N, NP). Inclusion of the three intermediate locations allows us to determine how the animals judge ambiguity in reference to the known rewarded and unrewarded locations. For instance, do they behave as though they anticipate a reward (a short approach latency: an optimistic response) or no reward (a long approach latency: a pessimistic response).

### Anxiety tests

All tortoises received the behavioural anxiety tests within two weeks of completing cognitive bias testing.

#### Novel object test

Each tortoise was exposed once to a novel object (a bead coaster) in the arena previously used for cognitive bias training/testing, and to which they were well habituated. The arena was visually divided into four quadrants, and at the start of each 5 min trial the subject was placed in the diagonally opposite quadrant to the novel object (Siviter et al. 2016), which was the same for all subjects. The object and arena were cleaned with disinfectant prior to each trial. Behaviours recorded were: latency (s) to move; head extension; and latency (s) to reach the novel object (i.e. when an individual first entered the same quadrant as the novel object (as defined by Siviter et al. 2016). Head extension appears to reflect emotional state in tortoises, with increased extension observed when animals are relaxed/confident (Moszuti et al. 2017). This was measured as the distance in cm of the tip of the nose from the front of the shell every minute using ImageJ (Rasband 1997), and calibrated using a known measurement (i.e. the length of the shell). Measurements of head extension were calculated as a proportion of tortoise size (using shell length) to account for differences in tortoise size. Due to the camera position and variability in whether the tortoise’s head was up or down from the horizontal, there may have been a degree of foreshortening when measuring head extension. However, because this would result in an underestimation, this therefore represents a conservative measurement.

#### Novel environment test

Novel Environment tests were carried out the day after the Novel Object tests. Each tortoise was placed once into an arena that had had various sensory aspects altered to heighten novelty (Moszuti et al. 2017), e.g. wall and floor coverings of varied texture, colour and pattern, for 10 min. Behaviours recorded were latency (s) to move and head extension, measured as in the Novel Object test, with the prediction that more relaxed/confident individuals would move sooner and extend their heads further.

### Data analysis

In the cognitive bias test, the multiple exposures to each of the locations were averaged (e.g. Burman et al. 2008). In the anxiety tests, head extension was measured at 60 s intervals throughout the test and an average (mean) head extension for the whole test was then calculated for each tortoise (e.g. Moszuti et al. 2017). We used a non-parametric Friedman’s Test to compare latencies between locations in the cognitive bias test, with ‘Location’ (P/NP/M/NN/N) as a within-subjects factor, and Wilcoxon Tests for post-hoc comparison. A Spearman’s rank test was used to investigate correlations between: (1) performance (i.e. latency to approach (s) in the cognitive bias task at testing (P/NP/M/NN/N) and behavioural responses in the concurrent anxiety tests; and (2) the same behaviours in the two anxiety tests (e.g. latency to move (Novel Object test) & latency to move (Novel Environment test). All analyses were conducted in SPSS Version 22.0.

## Results

### Cognitive bias task

**Fig. 2:**
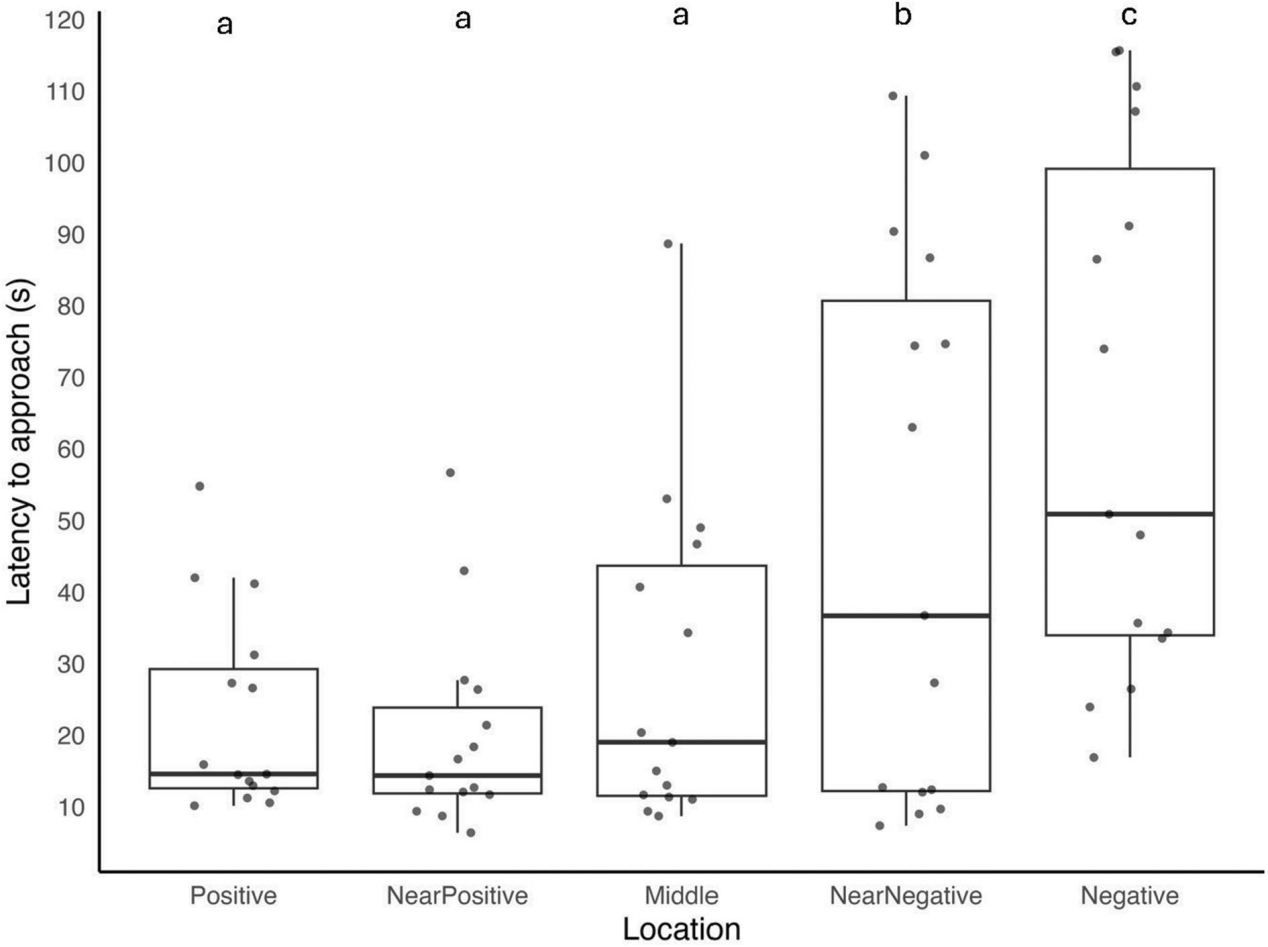
Time taken (in seconds) to reach the bowls in the testing phase in the five different locations for the cognitive bias task. Data are presented as medians and IQ range with individual data points. Letters (**a/b/c**) *when different* denote statistically significant post-hoc comparisons

All fifteen tortoises achieved criterion during training and were subsequently tested. There was a significant overall difference between the five different bowl positions (*N* = 15, ChiSquare = 25.493, df = 4, *p* < 0.001). Post-hoc pairwise comparisons revealed significant differences between N and all other positions (P: *p* = 0.001, NP: *p* = 0.001, M: *p* = 0.001, NN: *p* = 0.009) and between NN and all other positions (P: *p* = 0.017, NP: *p* = 0.012, M: *p* = 0.036, N: *p* = 0.009). There were no other significant positional differences (all *p* > 0.05) (see Fig. 2).

### Anxiety tests

#### Novel object test

**Fig. 3:**
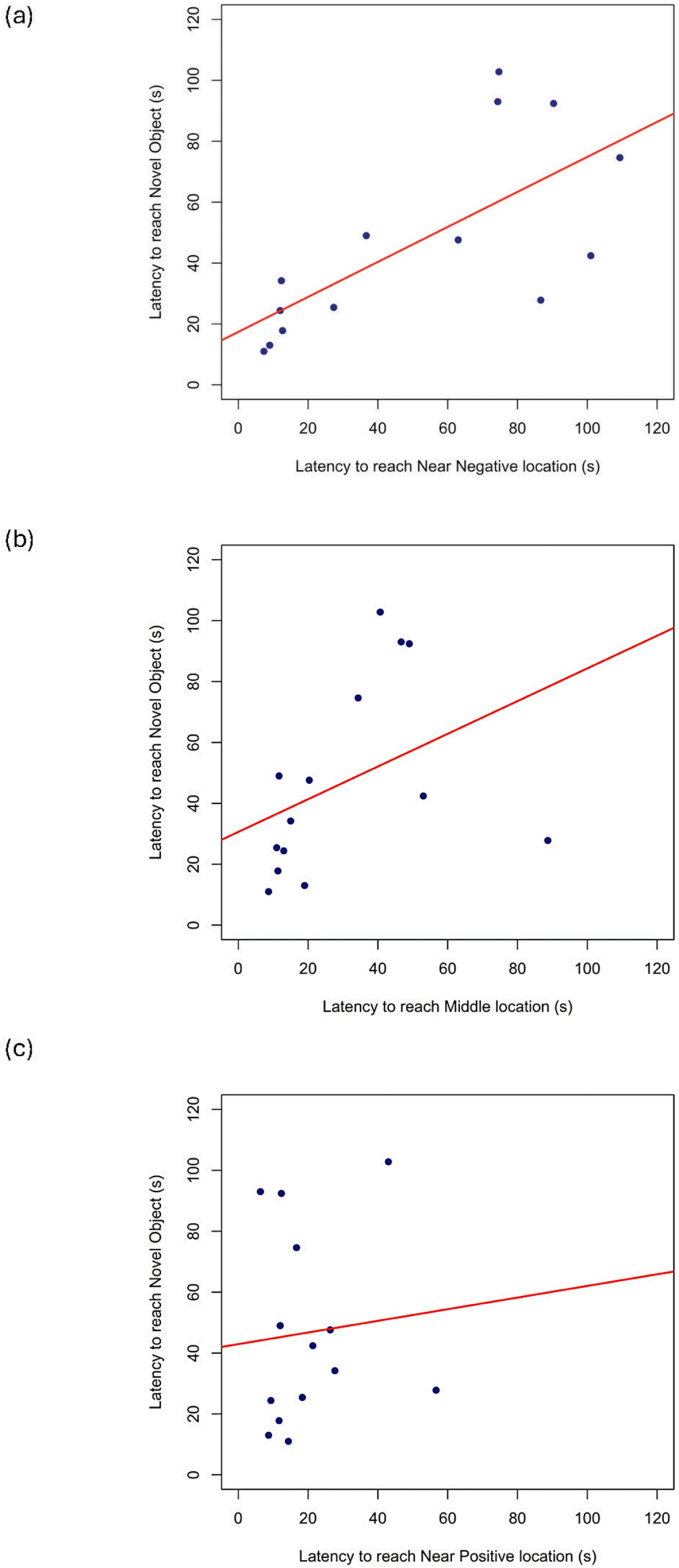
Latency to reach the object (s) (novel object test) and: (**a**) mean latency to reach the bowl in the Near Negative (NN) location (cognitive bias task); (**b**) mean latency to reach the bowl in the Middle (M) location (cognitive bias task); (**c**) mean latency to reach the bowl in the Near Positive (NP) location (cognitive bias task). All times are in seconds

One tortoise was excluded from the analysis due to defecation. There was a positive correlation between the latency to reach the novel object and the time taken to reach the bowl in the M (ρ = 0.591, *N* = 14, *p* = 0.026) and NN (ρ = 0.745, *N* = 14, *p* = 0.002) locations (see Fig. 3). No further significant correlations were found (all *p* > 0.05, See Table 1).

**Table 1:** Correlation between latency to reach the bowl in the three ambiguous locations (NP, M, NN) during cognitive bias testing and: latency to move, latency to reach the novel object and neck extension (as a proportion of body size) in the novel object test. Statistically significant results are in bold

Ambiguous Location (cognitive bias test)	Latency to Move	Latency to Reach Object	Neck Extension (adjusted by size)
NP	ρ=-0.003, *N* = 14, *p* = 0.993	ρ = 0.196, *N* = 14, *p* = 0.503	ρ = 0.174, *N* = 14, *p* = 0.553
M	ρ=-0.346, *N* = 14, *p* = 0.226	**ρ = 0.591**, *N*** = 14**, *p*** = 0.026**	ρ = 0.257, *N* = 14, *p* = 0.375
NN	ρ = 0.074, *N* = 14, *p* = 0.801	**ρ = 0.745**, *N*** = 14**, *p*** = 0.002**	ρ = 0.055, *N* = 14, *p* = 0.852

#### Novel environment test

A significant negative correlation was found between head extension and latency to reach the bowl in the NP position (ρ=-0.589, *N* = 15, *p* = 0.021). No further significant correlations were found (*p* > 0.05, See Table 2). We also found no significant correlations between behaviours in the two anxiety tests (all *p* > 0.05) with, for example, no significant correlation in latency to move (ρ=-0.22, *N* = 14, *p* = 0.939).

**Table 2:** Correlation between latency to reach the bowl in the three ambiguous locations (NP, M, NN) during cognitive bias testing and: latency to move; and neck extension (as a proportion of body size) in the novel environment test. Statistically significant results are in bold

Ambiguous Location (cognitive bias test)	Latency to Move	Neck Extension (adjusted by size)
NP	ρ = 0.493, *N* = 15, *p* = 0.062	**ρ=-0.589**, *N*** = 15**, *p*** = 0.021**
M	ρ=-0.006, *N* = 15, *p* = 0.982	ρ=-0.161, *N* = 15, *p* = 0.567
NN	ρ=-0.040, *N* = 15, *p* = 0.886	ρ=-0.154, *N* = 15, *p* = 0.585

## Discussion

We have provided the first evidence of the presence of a mood state in a reptile species as determined by performance in a cognitive bias task. This finding was reflected in associations between performance in the cognitive bias task and concurrent anxiety tests, with more optimistic individuals showing less anxious behaviour in response to novelty, and more pessimistic individuals showing behavioural indications of increased anxiety. These results significantly extend contemporary knowledge of the capacity for reptiles to experience mood states, and therefore have important implications not only for informing how reptiles are managed in captivity, but also for furthering our understanding of phylogenetic pathways of affective state and comparative species capabilities.

Our results suggest that reptiles can respond in a similar way to mammalian and avian species during a spatial cognitive bias task (Mendl et al. 2009), with individuals approaching ambiguous stimuli more rapidly when the stimuli were spatially closer to a rewarded location. There was also a general ‘optimistic skew’ towards judging ambiguous NP, M and P positions as collectively similar (Burman et al. 2009). This result challenges the view that, relative to mammals, reptiles might experience a comparatively narrow, non-egocentric, affective spectrum (Papini 2003), providing evidence that reptiles are capable of experiencing ‘free-floating’ mood states as well as stimulus-specific discrete emotions of both negative (e.g. emotional fever: Cabanac and Gosselin 1993; Cabanac and Bernieri 2000) and positive valence (e.g. pleasure: Balasko and Cabanac 1998). It would be valuable to extend this work to other reptile species to identify if variation exists within and between reptile clades.

In a study that used a similar correlational approach (Mendl et al. 2010), researchers found that dogs who scored highly in a test designed to measure separation-related behaviour, suggestive of an anxious emotional state, were also found to be more pessimistic in a cognitive bias task. Here, we observed similar associations between response in behavioural tests of anxiety (i.e. response to novelty) and cognitive bias task performance reflective of background mood. In our study, more optimistic individuals showed less anxious behaviour in response to novelty (e.g. they showed greater head extension in the novel environment), and more pessimistic individuals showed behavioural indications of increased anxiety (e.g. they showed an increased latency to approach the novel object). Furthermore, the lack of significant correlations between the latencies to approach the P or N ‘reference’ locations and performance in the anxiety tests indicates that the tortoises were not simply either fast or slow to move in general, but specifically in response to their judgement of ambiguity (Burman et al. 2009). This was confirmed by the negative association between latency to reach the bowl in the NP position and head extension — a measure that does not rely on locomotion. Perhaps surprisingly, we did not find a correlation between behaviours shown in the two separate tests of response to novelty. The assumption might be that both tests would generate a similar state of anxiety (i.e. due to the presence of novelty (either object or environment), but there is evidence to suggest that they could represent quite different emotional contexts (Stockley et al. 2020) with neophobia failing to be expressed similarly across contexts (Szabo and Ringler 2023).

To conclude, this study reveals that reptiles are capable of experiencing a far wider affective spectrum than previously considered, a finding that is critical in regards to how we view reptiles (e.g. in ascribing sentience) and the measures we take to ensure their welfare. If reptiles are capable of experiencing longer-term mood states as well as brief emotions then, taken alongside their greater-than-expected cognitive capabilities (Matsubara et al. 2017), we should assume that they are not only capable of suffering but also experiencing enduring positive moods, and we should therefore alter how we manage captive populations accordingly, such as by providing cognitive challenge through a complex and enriched environment (Hoehfurtner et al. 2021a, b; Rickman et al. 2025). We have also confirmed the efficacy of the cognitive bias approach, as well as behavioural measures of anxiety, for use in future studies to assess the impact of different housing and management practices on reptile welfare, thus extending the repertoire of valid assessment tools — a significant step forward for reptile welfare.

## Data Availability

Please email the authors for access to data and materials.
